# The cognitive and neural bases of human tool use

**DOI:** 10.3389/fpsyg.2014.01107

**Published:** 2014-10-06

**Authors:** François Osiurak, Cristina Massen

**Affiliations:** ^1^Laboratoire d'Etude des Mécanismes Cognitifs, Université Lyon 2Lyon, France; ^2^Institut Universitaire de FranceParis, France; ^3^Leibniz Research Centre for Working Environment and Human FactorsDortmund, Germany; ^4^Bonn-Rhein-Sieg University of Applied SciencesRheinbach, Germany

**Keywords:** tool use, action, motor control, neural bases, cognitive bases

It is a euphemism to say that humans use tools. Humans possess a vast repertoire of tools they use every day. In fact, as language or bipedal locomotion, tool use is a hallmark of humans. Tool use has also been often viewed as an important step during evolution (van Schaik et al., 1999) or even as a marker of the evolution of human intelligence (Wynn, 1985). So a fundamental issue is, what are the cognitive and neural bases of human tool use? The present series of papers in this special topic represents the newest additions to that research topic.

Central to that topic is the issue of the nature of the representations underlying tool use. Most of our understanding has come from the study of brain-damaged patients with tool use disorders, also called apraxia of tool use. When asked to light a candle, for example, those patients can light the candle correctly but then put it to the mouth in an attempt to smoke it. Such observations have led traditional cognitive models of apraxia to assume that tool use is supported by sensorimotor knowledge about tool manipulation (e.g., Rothi et al., 1991; Buxbaum, 2001). Consistent with this, Gainotti (2013) reviews a series of neuropsychological and neuroimaging studies indicating that perceptual, motor, and encyclopedic sources of knowledge have different weights in the construction of the different object categories (i.e., living things, tools) that are stored within the brain. This sensory-motor hypothesis assumes that manipulation knowledge stored within inferior fronto-parietal areas is critical to tool use skills. This link is also suggested by van Elk (2014), who conducted an fMRI study wherein participants had to predict the subsequent use of a presented tool. His results indicate that the left inferior parietal lobe might store hand-posture representations that can be used for planning tool-directed actions as well as for predicting other's actions.

Contrary to the traditional cognitive models of apraxia, a growing body of literature suggests that the left inferior parietal lobe might rather support technical reasoning, namely, the ability to reason about physical object properties (Goldenberg and Spatt, 2009; Osiurak et al., 2009, 2010, 2013; Goldenberg, 2013; Osiurak, 2014). Support for the technical reasoning hypothesis comes from findings demonstrating a strong association in left brain-damaged patients between the ability to use familiar tools and the ability to use novel tools to solve mechanical problems (for reviews, see Goldenberg, 2013; Osiurak, 2014). Four review articles of this special issue also provide evidence in line with the technical reasoning hypothesis. Bienkiewicz et al. (2014), Orban and Caruana (2014), and Vingerhoets (2014) emphasize that the ability to understand mechanical actions might be the specificity of the anterior portions of the inferior parietal lobe (particularly the supramarginal gyrus) while the posterior parietal cortex might be involved in the planning of the grasping and reaching components of both tool-use and non-tool-use actions. In the same vein, by reviewing studies investigating tool use disorders in left brain-damaged patients over the last 30 years, Baumard et al. (2014) suggest that the loss of mechanical knowledge might be the core deficit in left brain-damaged patients with apraxia of tool use.

Two experimental articles also address the issue of the involvement of mechanical vs. manipulation knowledge in tool use. First, Parry et al. (2014) examine both functional dynamics (i.e., the understanding of the mechanical actions involved in the task) and joint contribution profiles of participants with different levels of expertise in a primordial percussive task (i.e., production of stone flakes using the Oldowan method). Their results show that when people learn a tool use activity what they learn is the functional dynamics rather than any particular movement *per se*. Second, Müsseler et al. (2014) asked participants to use lever tools or to imagine using them in order to explore the role played in response generation by the spatial compatibility relationships between stimulus (S; at which the effect points of the lever aims at), responding hand (R) and effect point of the lever (E). They observed that the most prominent compatibility effects were for RE compatibility, corroborating the idea that even in tool use planning is influenced not only by the spatial relationship between stimulus and response, but also by the intended action effects. Similar results are reported by Rieger et al. (2014), who had participants perform circling movements with a stylus (movement) and presented distorted visual feedback of the movements on a screen (visual effect). When participants had to synchronize the visual feedback dot with a second, rotating stimulus on the screen (stimulus), strong compatibility effects emerged for the relationship between the hand movement (response) and the visual effect of this movement on the screen.

As Fagard et al. (2014) state, the development of tool use in human infants has received little interest until recently. For example, an unresolved issue is whether tool use appears through sudden insight or emerges progressively through familiarization with experience. Fagard et al. (2014) address this issue by conducting a longitudinal study on five infants from age 12 to 20 months. Children have to use a rake-like tool to reach toys presented out of reach. Their results indicate that it is only between 16 and 20 months that the infants suddenly start to intentionally try to bring the toy closer with the tool. For them, this sudden success at about 18 months might correspond to the coming together of a variety of capacities, such as the development of means-end behavior.

Tools are also specific because they modify our perception of the world. For instance, it is known that using a tool can alter space perception in that far stimuli become processed as if they were nearer (Maravita and Iriki, 2004; Witt et al., 2005; Osiurak et al., 2012). Likewise, body representations can be modified when using a tool so that the tool is incorporated and becomes part of our body (Iriki et al., 1996; Cardinalli et al., 2009). An interesting issue, however, is whether these modifications only occur after the real use of tools or can also appear in a tool-use imagery condition. Baccarini et al. (2014) provide a positive answer to this issue by showing that tool-use imagery is sufficient to affect the representation of the user's arm.

In line with the view of common representations for perception, imagery, and action, Kelly and Wheaton (2013) investigate the understanding of tool-use actions viewed from different perspectives and conclude that perception and understanding is facilitated when tool-use actions are viewed from an egocentric (as opposed to allocentric) perspective.

Finally, two theoretical papers also contribute to this special topic on broader issues. In line with the extended mind view, Borghi et al. (2013) suggest that words can be conceived as quasi-external devices (or tools) that extend our cognition. For example, words function like tools because they also enlarge the bodily space of action and, as a result, modify our sense of body. Baber et al. (2014) propose the notion of distributed cognition to account that tool use is not only based on internal representations (e.g., manipulation knowledge or mechanical knowledge) but also external representations such as the location of tools within the workspace.

In sum, this special issue includes a series of articles from neuropsychology, neuroimaging, experimental psychology, developmental psychology, and ergonomics that provide very interesting findings and open new issues for future research on the topic. Let's hope that we possess the good tools to solve them!

## Conflict of interest statement

The authors declare that the research was conducted in the absence of any commercial or financial relationships that could be construed as a potential conflict of interest.
